# Hepatitis B virus X protein promotes the growth of hepatocellular carcinoma by modulation of the Notch signaling pathway

**DOI:** 10.3892/or.2012.1620

**Published:** 2012-01-03

**Authors:** FAN WANG, HAIYAN ZHOU, YUZHEN YANG, XIUMEI XIA, QIAN SUN, JIN LUO, BIN CHENG

**Affiliations:** Department of Gastroenterology and Hepatology, Tongji Hospital, Tongji Medical College, Huazhong University of Science and Technology, Wuhan 430030, P.R. China

**Keywords:** HBx, HepG2, Notch, NICD, DAPT

## Abstract

Hepatitis B virus X protein (HBx) plays a crucial role in the development of hepatocellular carcinoma (HCC), however, little is known about the mechanism. Here, we investigated the relationship between HBx and Notch signaling in HepG2 cells after they were transfected with the HBx gene. It was found that HBx upregulated the expression of Notch-1, Jagged-1 and Hes-1 at the transcriptional level by binding to the Notch-1 intracellular domain, which is congruent with the observations of enhanced malignant biological activities of HBx-transfected HepG2 cells compared with normal HepG2 cells. However, while Notch signaling was blocked, the HBx-induced abnormalities were partially reversed. These findings suggest that HBx may promote the progression of HCC via the activated Notch pathway.

## Introduction

Hepatocellular carcinoma (HCC) is one of the most malignant tumors worldwide and Hepatitis B virus (HBV) has been identified as the most important risk factor for developing HCC (1–3). Of the four proteins translated by HBV, the X-gene product (HBx) has been most closely associated with the HCC pathogenesis (4). The correlation between HBx and HCC development has been extensively studied and the oncogenic roles of HBx include the following: activation of a variety of transcription factors such as nuclear factor-κB (NF-κB) (5), activator protein 1 (AP-1) (6), cAMP-responsive element binding protein/activating transcription factor 2 (CREB/ATF-2) (7); interaction with cellular oncogenes, such as Ras (8) and Src (9); regulation of cell apoptosis and the cell cycle by interacting with caspases, CDK, CKI, and survivin (10,11); and stimulation of cell signaling pathways, such as the Wnt (12), the Ras/MAPK (13), and the PI3K-Akt/PKB pathway (14).

Notch signaling is a highly evolutionarily conserved pathway which plays a pivotal role in regulating the development of organs and tissues by affecting cell proliferation, differentiation, apoptosis and stem cell maintenance (15). In mammals, the Notch family consists of four transmembrane receptors (Notch-1-Notch-4) and five ligands (Jagged-1, Jagged-2, Dll-1, Dll-3 and Dll-4) (16). Upon receptor-ligand binding, the Notch receptor is proteolytically cleaved by a γ-secretase complex (17), which results in releasing of the Notch intracellular domain (NICD) (18). Then NICD translocates into the nucleus and bands to the transcriptional factors known as CSL (CBF-1/suppressor of hairless/Lag-1), leading to the transcriptional activation of Notch target genes, including basic helix-loop-helix (bHLH) proteins such as HES-1 and HES-5 (19,20). Mounting evidence shows that perturbation of Notch signaling often leads to tumorigenesis (21–24). Some studies have shown the potential roles of Notch signaling in the development of HCC (25,26).

However, up to now, only a few reports about the role of Notch signaling in HBx-related HCC. In this study, we investigated the relationship of the Notch pathway with HBx in HepG2 cells, and found that HBx can enhance the progression of HCC via the activation of Notch pathway, which may provide some new clues for the potential role of Notch signaling in HBx-associated liver cancer.

## Materials and methods

### Cell culture

The human hepatoma cell line HepG2 were obtained from the American Type Culture Collection (ATCC; Manassas, VA, USA). The HepG2/HBx and HepG2/pcDNA3.1 cell lines, were derived from HepG2 cells by transfecting with HBx expression plasmid or an empty plasmid (pcDNA3.1(+)/V5-HisB), respectively. Both cell lines have been successfully established (27). All cell lines were cultured in DMEM (Gibco, Carlsbad, CA, USA) supplemented with 10% fetal bovine serum (FBS; Gibco, Grand Island, NY, USA) and maintained in humidified incubator at 37°C in a 5% CO_2_ atmosphere.

### DAPT treatment

The γ-secretase inhibitor N-[N-(3,5-difluorophenacetyl)-L-alanyl]-S-phenylglycine t-butyl ester (DAPT) was purchased from the Sigma-Aldrich Company (St. Louis, MO, USA). DAPT was dissolved in 100% dimethylsulphoxide (DMSO, Sigma) to make a stock solution of 10 mM, which was then diluted in culture medium to obtain the desired concentrations of 1, 5, 10 and 20 μM. DMSO diluted in culture medium at the final concentration of 0.05% without DAPT was designated as 0 μM. Untreated cells were those incubated in the culture medium without any additives. Cells treated with or without DAPT were cultured for 48 h, after which the total RNA or protein was extracted and flow cytometry was carried out.

### Cell proliferation and viability assays

Cell proliferation assays were performed by using a Cell Counting Kit-8 (Dojindo, Kumamoto, Japan) according to the manufacturer’s instructions. Briefly, 1×10^4^ cells/well were plated in 96-well plates and cultured with growth medium. At the indicated time points, the medium was aspirated. Then 100 μl serum-free DMEM and 10 μl WST-8 [2-(2-methoxy-4-nitrophenyl)-3-(4-nitrophenyl)-5-(2,4-disulfophenyl)-2H tetrazolium, monosodium salt] were added to each well followed by incubation at 37°C for 1.5 h. Absorbance was measured at 450 nm with a reference wavelength of 630 nm on a spectrophotometer (Molecular Devices, Sunnyvale, CA). To evaluate the viability of HepG2/HBx cells, 1×10^4^ cells/well were plated in 96-well plates followed 12 h later by the addition of DAPT at concentrations of 0, 1, 5, 10 or 20 μM. The cell viability was assessed as the percent of viable cells relative to the untreated control cells, which was determined for each concentration using the following equation: % viability = OD_experiment_/OD_control_ × 100%. Control cells were considered as 100% viable. All experiments were repeated five times.

### Cell cycle and apoptosis analysis by flow cytometry

After treatment with or without DAPT for 48 h, cells were harvested, immediately fixed in 75% ethanol at 4°C overnight, treated with 50 mg/l RNAse A (Sigma) for 30 min at 37°C, and stained with 50 mg/l PI (Sigma) for 10 min. Samples were then analyzed for their DNA content by a FACSAria Cell Cytometer (BD Biosciences, San Jose, CA, USA). The data were analyzed with the CellQuest software (BD Biosciences). Apoptosis analysis was performed by using a Annexin-V-FITC kit (Bender MedSystems, Burlingame, CA, USA) according to the manufacturer’s instructions. The percentage of cells that were Annexin-V positive but PI negative was compared among the different treatment groups.

### Immunofluorescence assays

Immunofluorescence assays were performed as previously described (28). HepG2/HBx cells were cultured on glass coverslips for 24 h and fixed with 4% paraformaldehyde. The fixed cells were incubated with anti-HBx (1:200, Santa Cruz Biotechnology, Santa Cruz, CA) and anti-NICD (1:200, Santa Cruz Biotechnology) for 12 h at 4°C, then incubated with Cy3-conjugated goat anti-mouse IgG (1:100, Boster, China) and FITC-conjugated goat anti-rabbit IgG (1:100, Boster) for 1 h. Nuclei were stained with diamidinophenyl indole (DAPI) (Boster). The stained cells were observed using a Fluoview FV1000 laser scanning confocal microscope (Olympus, Japan).

### Co-immunoprecipitation assays

Co-immunoprecipitation assays were performed as previously described (28). HepG2/HBx cells were lysed, and the lysates were pretreated with protein G-agarose (Santa Cruz Biotechnology) to remove non-specifically bound proteins. After centrifugation, one third of the supernatants were stored at −80°C as a positive control. The remaining supernatant samples were incubated for 2 h at 4°C with 1 μg of non-immune mouse IgG or mouse anti-HBx (Santa Cruz Biotechnology), for the negative control and the experimental group, respectively. Then, the mixture was incubated for 1 h to overnight at 4°C with 20 μl protein G-agarose (Santa Cruz Biotechnology). The immunocomplexes were extensively washed with PBS, samples were boiled in electrophoresis sample buffer and assayed by Western blot analysis.

### Real-time PCR analysis

Total RNA was isolated from cultured cells using TRIzol reagent (Invitrogen, Carlsbad, CA, USA) and cDNA was synthesized from 2 μg of total RNA using Moloney murine leukemia virus reverse transcriptase (MMLV) (Promega, Madison, WI, USA). Real-time quantitative PCR (qRT-PCR) using SYBR Premix (DRR041A, Takara, Japan) was performed as previously described (29). Amplifications were performed in a LightCycler machine (Roche Diagnostics, Basel, Switzerland) following the manufacturer’s instructions. The HepG2 cDNA was used as standard cDNA. A standard curve for each gene was generated from serially diluted standards, and values for unknown samples were extrapolated. β-actin was used as an internal control to normalize samples. All standards and samples were assayed in triplicate. The primer sequences used to amplify specific target genes are listed in Table I.

### Western blot analysis

Cells were lysed as previously described (29) and the lysates were subjected to electrophoresis on SDS-PAGE and transferred to PVDF membranes (Millipore, Billerica, MA). The blotted membranes were blocked and subsequently incubated with rabbit anti-Jagged-1 (1:600), rabbit anti-Notch-1 (1:1,000), rabbit anti-Notch-1-IC (1:1,000), rabbit anti-Hes-1 (1:600), and rabbit anti-actin (1:1,000). All antibodies were from Santa Cruz Biotechnology except for anti-Notch-1-IC which was obtained from Cell Signaling Technology (Danvers, MA). After incubation with horseradish peroxidase-labeled secondary antibody (1:5,000–10,000; Santa Cruz Biotechnology), visualization was performed by an enhanced chemiluminescence kit (Pierce, Rockford, IL) and exposure to X-ray film (Kodak, Rochester, NY). Immunoblotting with the anti-actin antibody was used as an internal control to confirm equivalent protein loading. Each experiment was performed at least 3 times. The relative intensity of each protein band was scanned by Quantity One software (Bio-Rad Laboratories, Hercules, CA, USA).

### Statistics

SPSS version 17.0 software (SPSS for Windows, Inc., Chicago, IL, USA) was used for all statistical analyses. All results are expressed as mean ± SEM. Statistical analysis of the data was performed using a standard one-way ANOVA or one-way ANOVA for repeated measures, followed by the least significant difference (LSD) post-hoc test. Bonferroni’s correction was used to adjust for multiple comparisons. A 2-tailed Student’s paired t-test was also used to compare the differences in the values between two groups. A P-value <0.05 was considered to be statistically significant.

## Results

### The Notch signaling pathway is activated in HepG2/HBx cells

**Figure 1:**
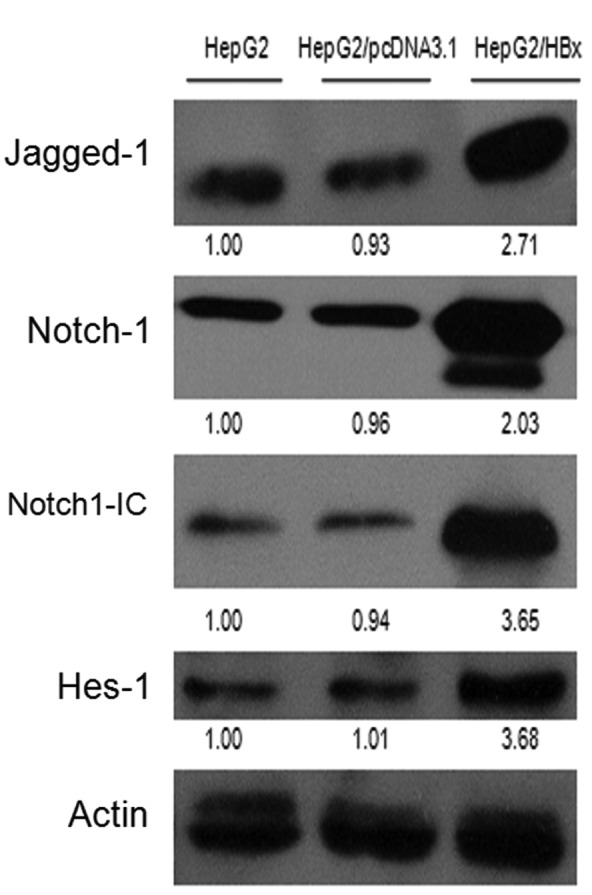

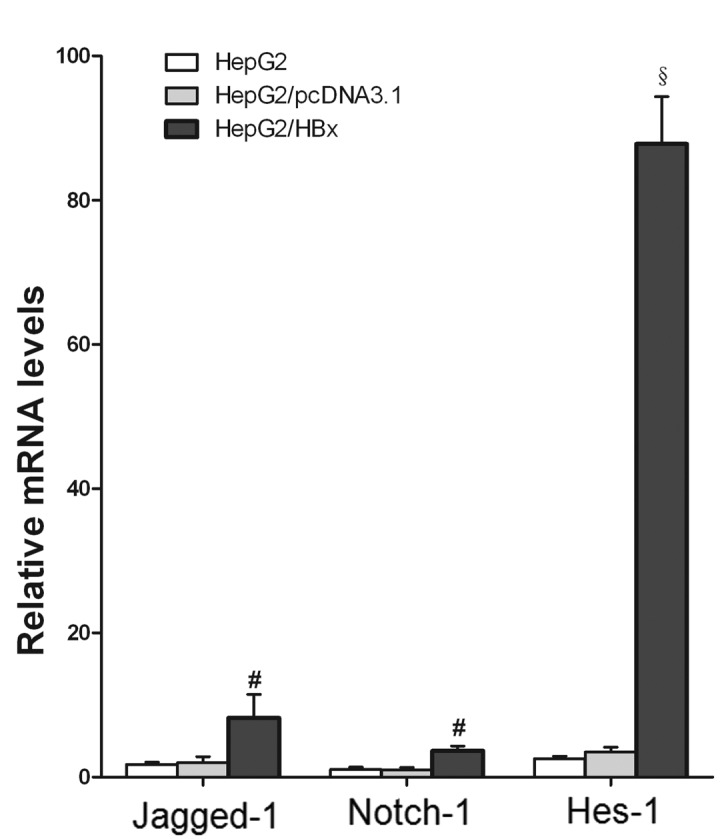

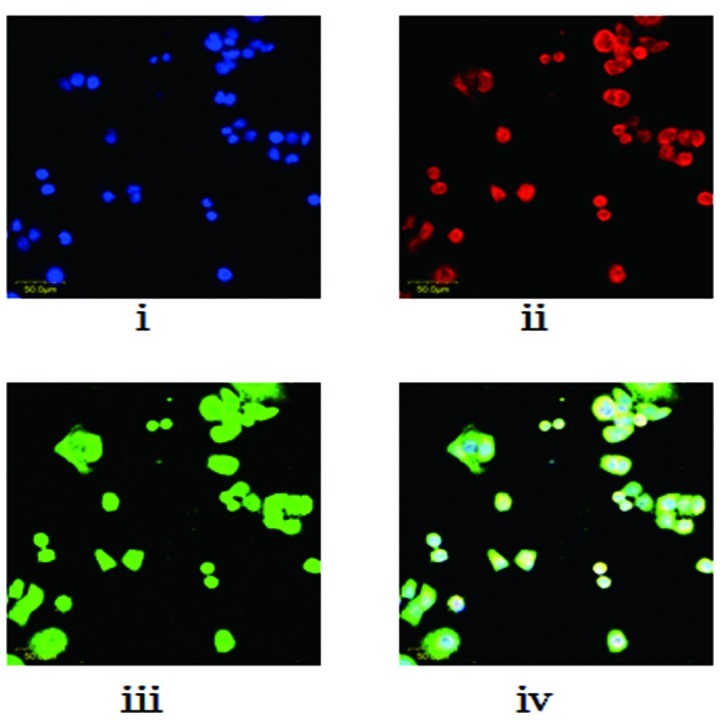

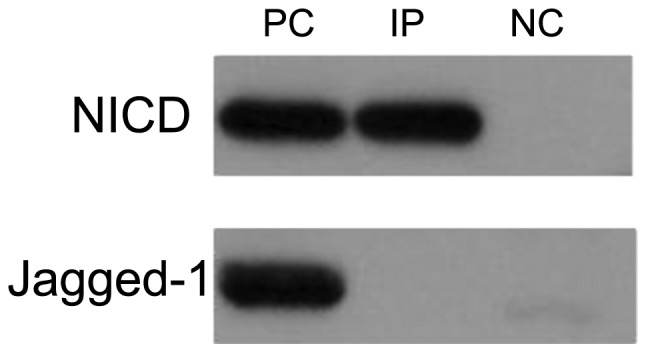
Expression of Notch signaling pathway components and the physical interaction of NICD with HBx in HBx-expressing cells. (A) The protein levels of Notch signaling pathway components were detected using western blot analysis. Actin was used as an internal control for equal loading of samples. Representative blots shown were from three independent experiments with identical results. The relative ratios of each band were normalized to actin and are shown below each western blot band. (B) Jagged-1, Notch-1, Hes-1 mRNA expression levels were determined by quantitative real-time RT-PCR. Results are mean ± SEM of 3 independent experiments. ^#^P<0.01 and ^§^P<0.001 vs. control (HepG2 cells). (C) Confocal microscopy imaging of the co-localization of NICD with HBx in L02/HBx cells. The nuclei were stained with DAPI (blue) (i), HBx protein was stained with Cy3-conjugated goat anti-mouse IgG (red) (ii) and the protein of NICD was stained with FITC-conjugated goat anti-rabbit IgG (green) (iii). Yellow staining indicates overlapping areas of red and green fluorescent labels (iv). (D) Co-immunoprecipitation assay of the interaction of NICD and Jagged-1 with HBx in HepG2/HBx cells. The preparation of proteins was described in Materials and methods. All the proteins were analyzed by western blot analysis using anti-NICD, anti-Jagged-1. The data shown are representative of 3 independent experiments. PC, positive control; IP, co-immunoprecipitation of proteins treated with anti-HBx; NC, co-immunoprecipitation of proteins treated with non-immune mouse IgG (negative control).

Previously, we observed that the HBx protein stimulated the proliferation as well as the cell cycle, inhibited the apoptosis of HepG2 cells significantly, and promoted tumour growth in nude mice (27). To investigate the potential involvement of Notch signaling in the development of HCC, we performed western blot analyses in HepG2, HepG2/pcDNA3.1 and HepG2/HBx cells. As shown in Fig. 1A, the protein levels of Jagged-1, Notch-1, Notch-1C and Hes-1 in HepG2/HBx cells were elevated relative to the controls. Then, we analyzed the mRNA levels of Jagged-1, Notch-1 and Hes-1 using quantitative real-time RT-PCR (qRT-PCR) and found that their mRNA levels were significantly increased in HepG2/HBx cells as compared with the controls (Fig. 1B).

### Notch-1 colocalizes and interacts with HBx in HepG2/HBx cells

To study the possible relationship between Notch-1 and HBx, we performed immunofluorescence and co-immunoprecipitation assays. As shown in Fig. 1C, the nuclei were stained blue (i), HBx was stained red (ii) whereas the NICD was stained green (iii) in HepG2/HBx cells. Yellow staining in the dual-labeling experiments indicates overlapping areas of red and green fluorescent labels (iv), suggesting co-localization of NICD with HBx in the HepG2/HBx cells. The compounds immunoprecipitated with anti-HBx or non-immune mouse IgG from HepG2/HBx cells were subjected to western blot analysis with anti-NICD and anti-Jagged-1, respectively. As shown in Fig. 1D, NICD was co-immunoprecipitated with HBx. No specific interaction was found between Jagged-1 and HBx or the protein immunoprecipitated by non-immune IgG, indicating the specificity of the NICD-HBx interaction.

### Inhibition of Notch signaling attenuates the growth of HepG2/HBx cells

**Figure 2:**
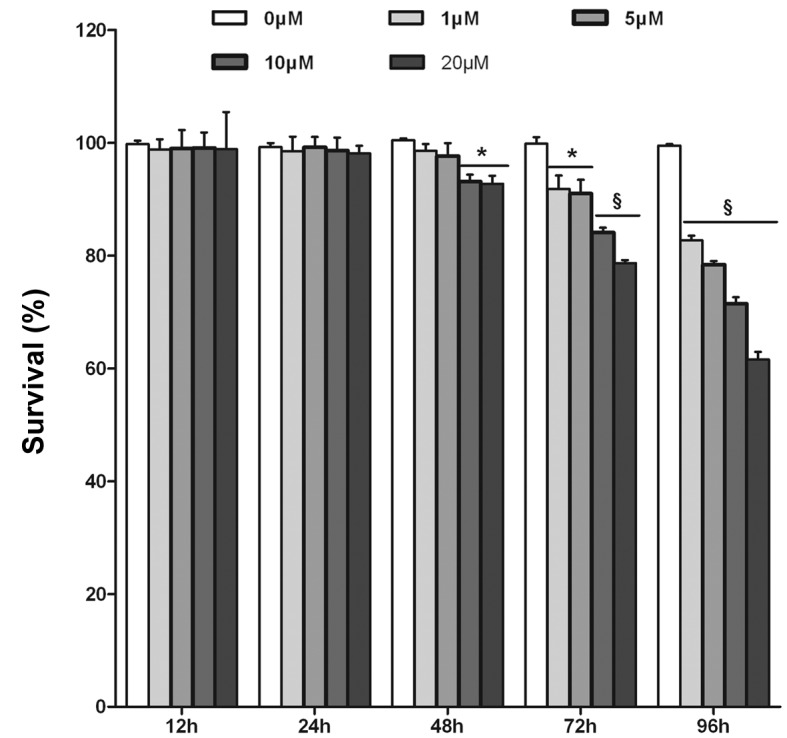

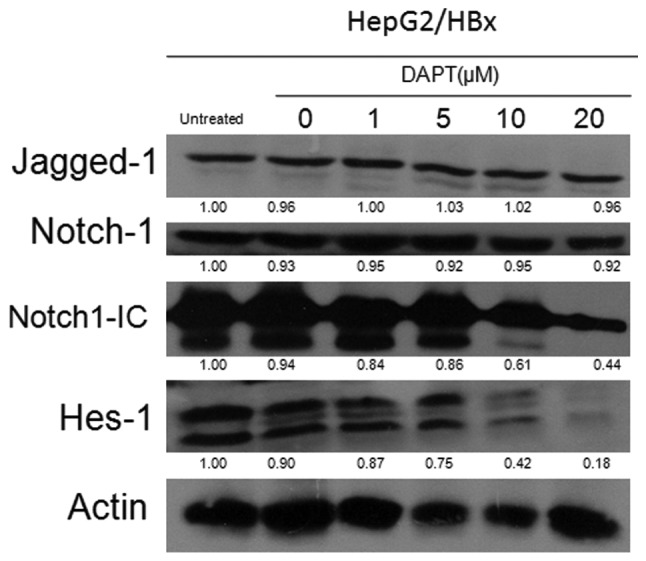

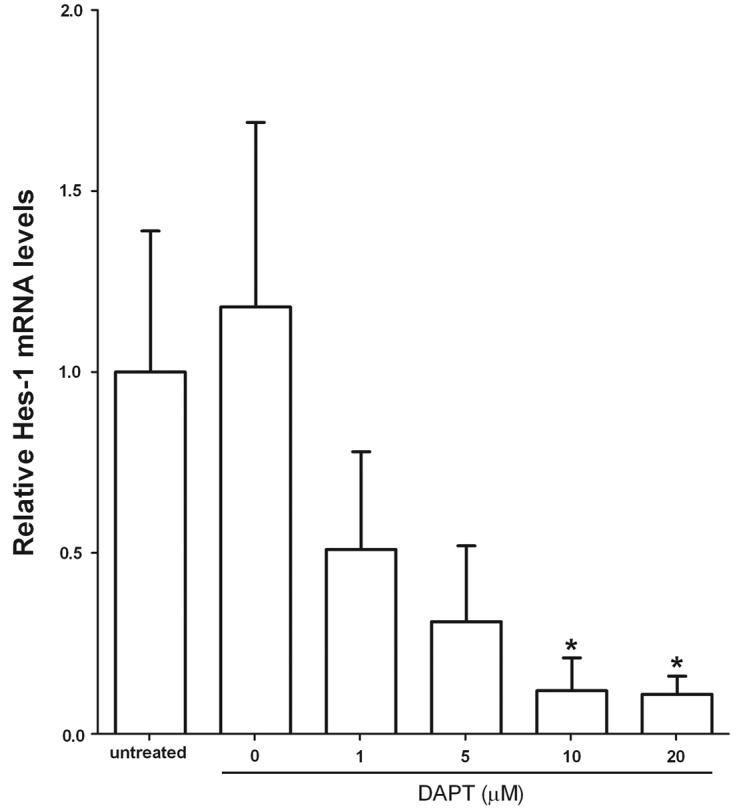
(A) Effects of inhibition of Notch signaling on the growth of HepG2/HBx cells. The effect of DAPT on cell viability of HepG2/HBx cells was determined by the WST-8 assay as described in Materials and methods. Results are expressed as mean ± SEM of five samples. ^*^P<0.05 vs. control (non-DAPT); ^§^P<0.001 vs. control. (B and C) Notch signaling in HepG2/HBx cells by the γ-secretase inhibitor DAPT. (B) Inhibition of Notch signaling in HepG2/HBx cells with indicated concentrations of DAPT was assessed by immunoblotting. Cells were harvested after 48 h of treatment. Actin was used as an internal control for equal loading of samples. Representative blots shown are from three independent experiments with identical results. The relative ratios of each band were normalized to actin and are shown below each western blot band. (C) Hes-1 mRNA levels were assessed by qRT-PCR in DAPT-treated HepG2/HBx cells. Cells were harvested after 48 h of treatment. Data shown are mean ± SEM of representative experiment performed in triplicate. ^*^P<0.01 vs. control (untreated cells).

In order to further establish that active Notch signaling is important for HBx to function as an oncoprotein, we treated HepG2/HBx cells with various concentrations of DAPT for 12 to 96 h and assessed cell proliferation by the WST-8 assay. As shown in Fig. 2A, in HepG2/HBx cells, increasing concentrations and durations of treatment with DAPT resulted in a decrease of cell viability. In contrast, treatment with various concentrations of DAPT for 12 or 24 h did not produce any significant reduction in cell viability. However, treatment of DAPT for >48 h resulted in significant dose- and time-dependent reduction in cell viability of HepG2/HBx cells. A significant reduction in cell viability by DAPT treatment was observed at concentrations of 10 and 20 μM after 48 h, with inhibition rates of 7.30 and 7.73%, respectively (P<0.05, Fig. 2A). We, therefore, selected the DAPT treatment time point of 48 h for further studies.

### Confirmation of the inhibition of Notch signaling in HepG2/HBx cells

HepG2/HBx cells treated with various concentrations of DAPT for 48 h and assessed for the DAPT inhibitory effects on Notch-1 signaling by western blot analysis of Jagged-1, Notch-1, Notch-1-IC, Hes-1 protein levels, and by qRT-PCR of Hes-1 transcripts. As shown in Fig. 2B and C, DAPT treatment greatly reduced the amount of Notch-1-IC and Hes-1 protein in a dose-dependent manner, while there was no significant effect on Jagged-1 and Notch-1 levels (Fig. 2B). A significant reduction of Notch-1-IC and Hes-1 protein levels were observed at 10 and 20 μM of DAPT treatment. qRT-PCR experiments showed that the downregulation of Hes-1 transcripts in HepG2/HBx cells occurred after treatment with DAPT at 10 (P<0.05) and 20 μM (P<0.05) (Fig. 2C). These data were consistent with the previous results that treatment with 10 and 20 μM DAPT for 48 h significantly inhibited the proliferation of HepG2/HBx cells.

### Inhibition of Notch signaling arrests G0/G1 phase, shortens the S phase and induces apoptosis in HepG2/HBx cells

**Figure 3:**
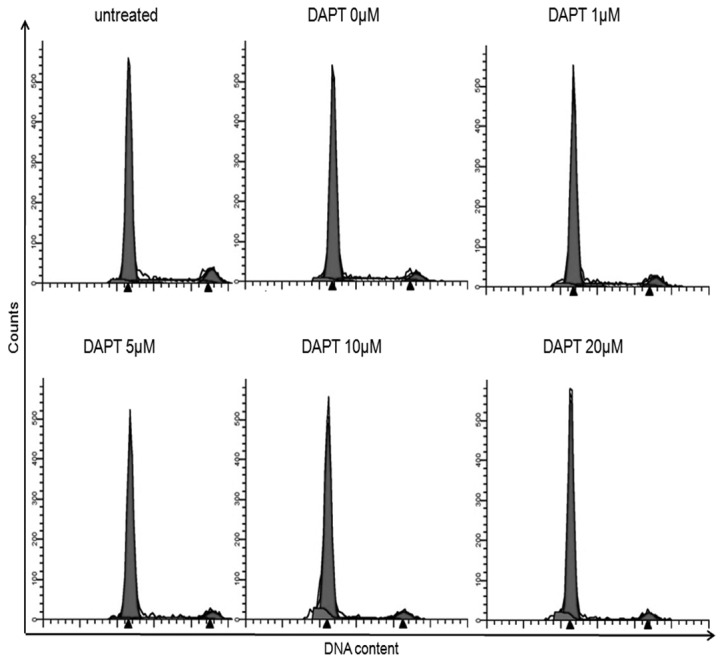

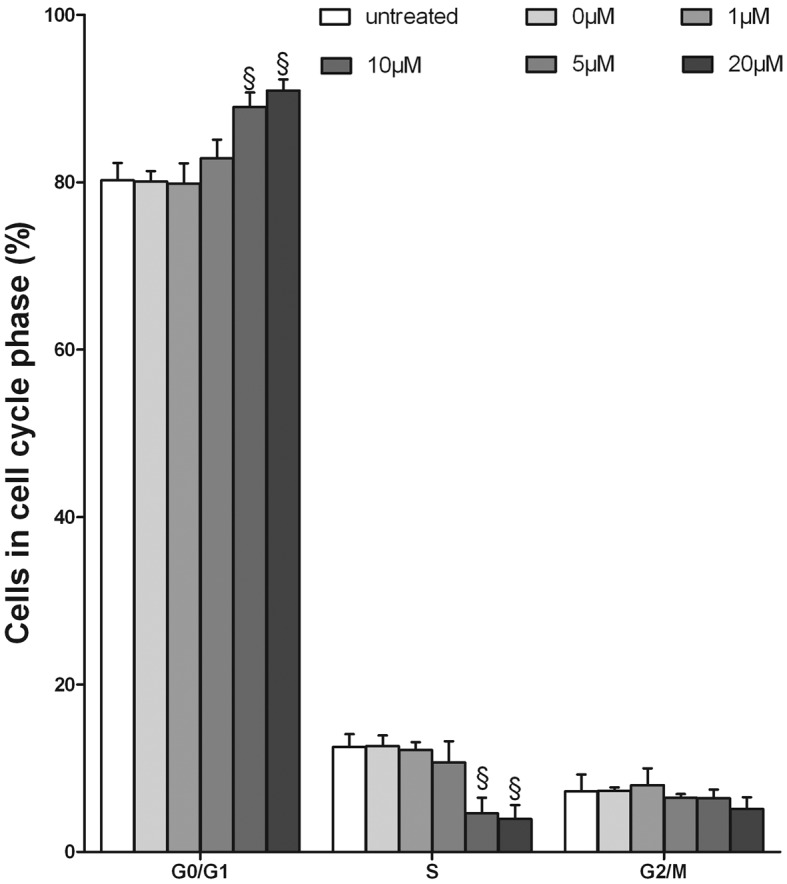

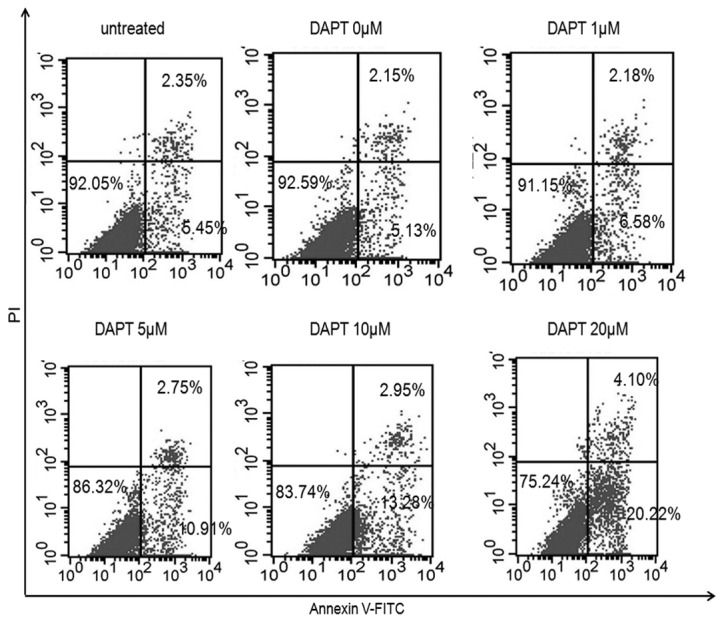

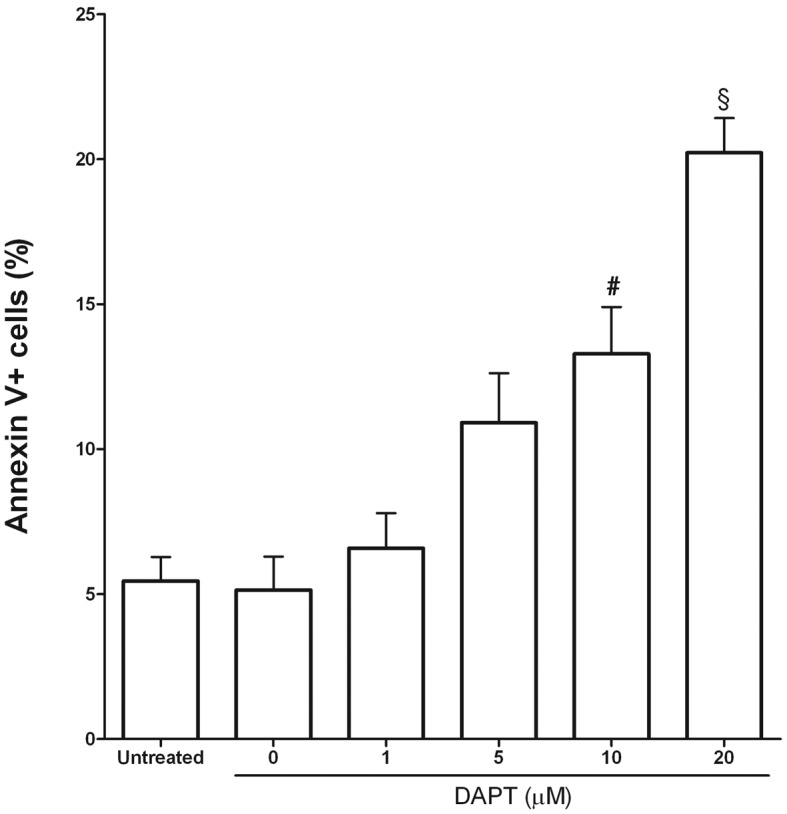
Effects of the inhibition of Notch signaling in G0/G1 distribution, and apoptosis in HepG2/HBx cells. (A and B) The cell-cycle distribution of HepG2/HBx cells was examined by PI staining and flow cytometry synchronized by serum starvation, and treated with or without DAPT for 48 h. Results shown are from a representative experiment (A) or mean ± SEM of 3 experiments (B), each performed in triplicate. ^§^P<0.05 vs. control (untreated cells). (C and D) Apoptosis of HepG2/HBx cells treated with or without DAPT for 48 h detected by Annexin-V and PI staining using FACS analysis. The percentage of cells that were Annexin-V positive, but PI negative were compared among different groups. Results shown are from a representative experiment (C) or mean ± SEM of 3 experiments (D), each performed in triplicate. ^#^P<0.01 and ^§^P<0.001 vs. control (untreated cells).

We performed PI staining and flow cytometry to define the cell-cycle distribution of DAPT-treated HepG2/HBx cells. Treatment of HepG2/HBx cells with 0–20 M of DAPT for 48 h resulted in arrest in the G0/G1 phase and shortening of the S phase. The proportion of HepG2/HBx cells in the G0/G1 phase was significantly increased at concentrations of 10 (88.99%, P<0.05) and 20 μM (90.95%, P<0.05), compared to that of 80.23% in untreated control cells (Fig. 3A and B). The proportion of cells in S phase was significantly decreased at concentrations of 10 (4.60%, P<0.05) and 20 μM (3.93%, P<0.05), relative to that of 12.55% in untreated control cells (Fig. 3A and B). No significant difference was observed between the G2/M phases. As shown in Fig. 3C and D, the apoptotic rates were increased from 5.45% in non-DAPT-treated cells to 13.28% at a concentration of 10 μM (P<0.01, Fig. 3C and D) and to (20.22%) at 20 μM (P<0.001, Fig. 3C and D) after treatment with DAPT for 48 h. Taken together, these results indicate that the inhibition of Notch signaling was associated with decreased DNA synthesis (S phase) and increased apoptosis, which contributed to the impaired growth of HepG2/HBx cells treated with DAPT.

### HepG2 cells are not significantly affected by Notch inhibition

**Figure 4:**
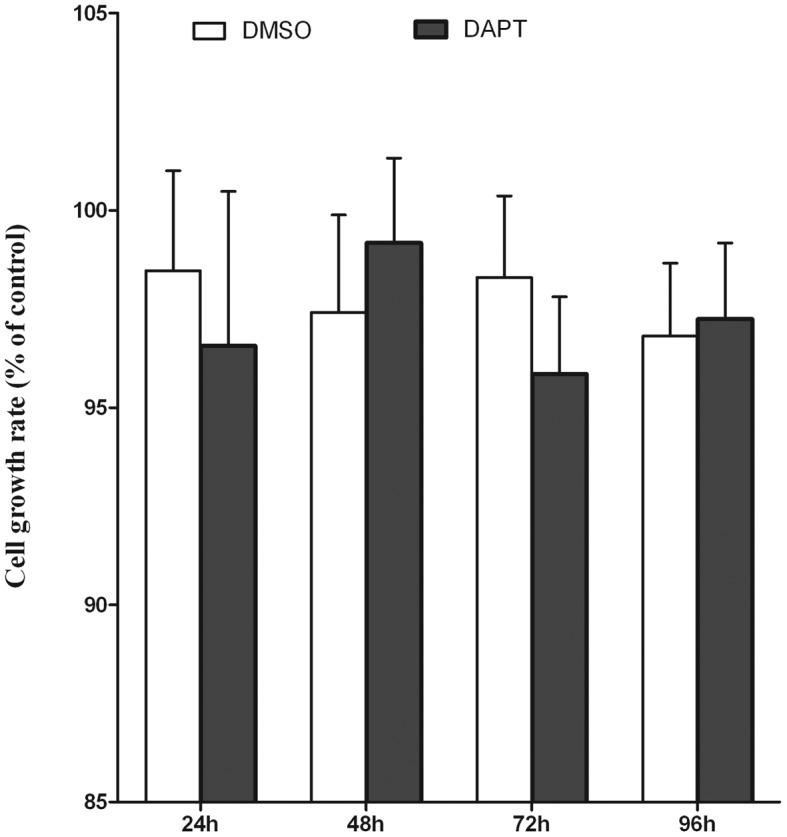

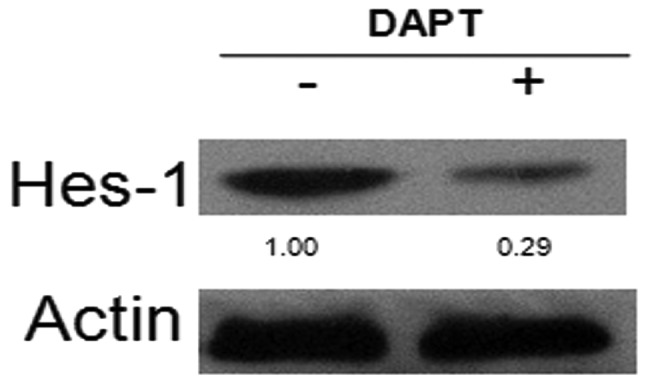
Inhibition of Notch signaling in HepG2 cells. HepG2 cells were treated with DAPT at the final concentration of 10 μM or with 0.05% DMSO. (A) HepG2 cells (1×10^4^) were cultured in medium contained DAPT or DMSO for indicated times. Cell growth was assessed by the WST-8 assay as described in Materials and methods and plotted as a percentage of that of the control (untreated cells). Bars represent the means ± SEM of each time point from five samples. (B) HepG2 cells were harvested after 48 h of treatment with DAPT (10 μM) or vehicle control (0.05% DMSO). Inhibition of Notch signaling in HepG2 cells was monitored by immunoblotting for Hes-1 protein levels (+, DAPT; −, vehicle control).

As a control, Notch signaling was blocked in HepG2 cells using DAPT as well. HepG2 cells were treated with DAPT at the final concentration of 10 μM or with 0.05% DMSO. The proliferation rate of HepG2 cells was measured after DAPT treatment using WST-8 assay. No effect on cell proliferation could be observed in HepG2 cells upon ablation of Notch signaling (Fig. 4A). The protein expression level of Hes-1 was monitored using western blot analysis. As shown in Fig. 4B, treatment of HepG2 cells with DAPT led to a distinct Hes-1 downregulation after 48 h. These results demonstrated that Notch signaling was much less important for the growth of HepG2 cells than for that of HepG2/HBx cells.

## Discussion

We previously reported that HBx protein stimulated the proliferation as well as cell cycle, inhibited the apoptosis of HepG2 cells significantly, and promoted tumor growth in nude mice (27). However, little is known about the mechanism of HBx action. A large number of studies have demonstrated that Notch signaling plays a crucial role in various malignant tumors (21–24). Expression and localization of Notch receptors and their ligands have been observed in the normal human liver tissue and the deregulated Notch signaling had been found in malignant liver tumors (25,26).

To examine whether Notch signaling was involved in the HBx-related HCC, we investigated the relationship between HBx and Notch signaling in HepG2 cells after being transfected with the HBx gene. It was found that HBx protein upregulated the expression of Notch-1, Jagged-1 and Hes-1 at the transcriptional level, which is related with the stimulated growth of HepG2 cells by HBx *in vitro* and *in vivo*. However, when Notch signaling was blocked with a γ-secretase inhibitor DAPT, the inhibited Notch signaling decreased the proliferation, arrested the G0/G1 phase and shortened the S phase of the cell cycle and induced apoptosis in HepG2/HBx cells. It seemed that when Notch signaling was blocked, the abnormality caused by HBx in HepG2 cells was partially reversed. However, the inhibition of Notch signaling in untransfected HepG2 cells had barely any effect on their growth. Moreover, we found that HBx colocalized and interacted with NICD (Notch intracellular domain) in HepG2/HBx cells. These findings demonstrated that HBx can activate Notch signaling by binding to NICD, which may contribute to the stimulated growth of HepG2 cells both *in vitro* and *in vivo*. Considering that the HepG2 cell line was originated histologically from human hepatocellular carcinoma, our studies suggest that HBx can promote the progression of HCC via the activated Notch pathway.

In summary, our results demonstrate that HBx can act as an oncogenic factor to promote the progression of HCC by binding to NICD to activate the Notch signaling pathway, which may provide a new clue for the potential role of Notch signaling in the HBx-associated human liver carcinoma.

## Figures and Tables

**Table I:** Primer sequences for real-time polymerase chain reaction analysis.

Gene	Primer sequence	PCR product size (bp)	GenBank accession no.
Jagged-1	F: 5′-CAACACGGTCCCCATCAAG-3′		
	R: 5′-TACTTCAGAATTGTGTGTCCTTATTTTAGA-3′	76	NM_000214.2
Notch-1	F: 5′-CCGCAGTTGTGCTCCTGAA-3′		
	R: 5′-ACCTTGGCGGTCTCGTAGCT-3′	109	NM_017617.3
Hes-1	F: 5′-GCTAAGGTGTTTGGAGGCT-3′		
	R: 5′-CCGCTGTTGCTGGTGTA-3′	122	NM_005524.2
β-actin	F: 5′-GTTGCGTTACACCCTTTCTTG-3′		
	R: 5′-GACTGCTGTCACCTTCACCGT-3′	157	NM_001101.3

F, forward; R, reverse.

## Abbreviations

HCC: hepatocellular carcinoma
HBV: hepatitis B virus
HBx: HBV X protein
Notch-IC or NICD: Notch intracellular domain
CSL: mammalian CBF1, Drosophila Su(H), *C. elegans* LAG-1
DAPT: γ-secretase inhibitor N-[N-(3,5-difluorophenacetyl)-L-alanyl]-S-phenylglycine t-butyl ester
qRT-PCR: quantitative real-time RT-PCR
